# Attitudes regarding a warranty and the expected longevity of dental treatment amongst New Zealand dentists, dental students, and patients: a mixed methods survey

**DOI:** 10.1186/s12903-024-03860-3

**Published:** 2024-01-13

**Authors:** Belinda Liu, David Roessler, Zac Morse

**Affiliations:** 1 Faculty of Dentistry, Division of Health Sciences, University of Otago, PO Box 75661, Manurewa, Auckland 2243 New Zealand; 2 Department of Oral Health, Faculty of Health and Environmental Sciences, Auckland University of Technology, Auckland, 0627 New Zealand

**Keywords:** Dental guarantee, Dental warranty, Failed treatment, Longevity estimates, Patient expectation, Remediation

## Abstract

**Objectives:**

To investigate and compare estimates of the longevity of dental treatment, expectations for free remedial treatment, and attitudes about formal dental warranties among dentists, students, and patients.

**Materials and methods:**

This is a mixed-method cross-sectional questionnaire survey with convenience sampling from dentists, dental students, and patients in New Zealand. A questionnaire was distributed to New Zealand dentists (*n* = 28) and final-year dental students (*n* = 27). A separate questionnaire was provided to patients in a university dental clinic (*n* = 43). Mann-Whitney U, Chi-square and Pearson Correlation, and Binary logistic regression tests were used to test for differences between groups and correlations amongst variables. Qualitative data were analysed thematically.

**Results:**

Dentists believed that their posterior composite resin restorations would last longer (*p* = 0.014), would remediate failed crowns for longer (*p* = 0.002) and would provide longer crown warranties (*p* = 0.003) compared to students. Patients had higher expectations for restoration longevity and free remediation for failed treatment. Students were generally more willing to provide warranties. Crowns were perceived to be the most warrantable, while endodontic treatment was the least warrantable. Recall attendance, mechanical failure, and adequate oral hygiene were commonly proposed as warranty conditions for restorations and crowns. There was little consensus about complete dentures and endodontic treatment.

**Conclusions:**

There are significant disparities between the expectations of patients and clinicians regarding treatment longevity and free remediation times. Clinicians, in general, are willing to provide free remediation within a specified time frame, except for endodontic treatment, but are hesitant to provide formal dental warranties.

**Supplementary Information:**

The online version contains supplementary material available at 10.1186/s12903-024-03860-3.

## Introduction

Despite advances in modern materials and techniques, dental treatments will fail. Direct restorations and crowns fail for various reasons, and replacements constitute a larger proportion of treatment provided than the initial placement of restorations [1]. For example, 5% of newly made dentures are replaced within two years, and 15% of root canal treatments require re-intervention or extraction within three years [2, 3]. Dental treatment that fails within a short time frame is a financial burden on the patient, dentist or public health system.

Like other paid goods and services, dental patients will understandably want assurances and pose the question: “how long will this last?” Unfortunately, the answer to this question is seldom straightforward and not directly addressed in the literature. There are a number of important parameters, which include: the average longevity, CL_50_ (clinical longevity time where 50% of treatments have failed), annual failure and success rates. However, none of these measures can predict how long a dental treatment will last.

Some countries’ public health and insurance systems have policies regarding quality assurance in dentistry and remediation for failed treatment. A recommendation was made to the United Kingdom’s National Health Service in 2009 that the free replacement period for restorations should be three years, with the burden of cost on the provider [4]. In Mumbai, India, a social enterprise (Swasth) provides dental warranties of up to three years, provided patients come for preventative check-up appointments, which are more frequent for smokers. Anecdotally, they have found that the warranty incentivised patients to opt for more expensive treatment rather than extractions, access timely care and improve oral hygiene habits [5]. The Swedish guarantee insurance system covers most public and private dentists. For prosthetic treatment, retreatment is covered for two years for fixed prosthodontics and one year for removable prosthodontics [6, 7]. However, there is little formal longevity assurance for patients in private practice globally, including in New Zealand. Failed dental treatment is often repaired or replaced within a poorly defined timeline at the clinician’s discretion. A formal limited warranty in private dentistry is an emerging concept, but currently with poor uptake.

Hopenwasser (2017) concluded that, since most practices are already providing replacement work for free, “why not make it into a positive marketing bonus?” [8]. This practice believes their warranty improves patient confidence and helps their recall program. Various practices in different countries, including New Zealand, advertise warranties on their websites, ranging from one to three years for composite resin restorations, up to five years for crown and bridgework, up to three years for dentures and five years for endodontic treatment. However, after a comprehensive review of the literature and to the authors’ knowledge, there are no empirical studies regarding dental warranties.

Therefore, the rationale for this study was to provide data for the underinvestigated topic of dental warranties, which could be useful in informing practice and policy. The aims were to investigate and compare longevity estimates for dental treatment, expectations for free remedial treatment, and attitudes toward a formal dental warranty between dentists, students, and patients.

## Materials and methods

This was a mixed-method cross-sectional survey using convenience sampling for participant recruitment. General dental practitioners were recruited at a monthly branch meeting of the Auckland Dental Association. Inclusion criteria included general dental practitioners who were currently in practice. Retired dentists, specialist dentists, and dentists who were practising in a purely academic or educational setting were excluded. All [27] dental students who were undertaking their final year at the University of Otago, Auckland Dental Facility, were recruited. Patients aged 18 and over were recruited from the reception area of the Otago University, Auckland Dental Facility. Both new and existing patients were invited to complete the survey while awaiting their appointment in the reception area. Patients with experience working in the dental field were excluded from the survey. Patients were not excluded based on oral or general health status. As this was an exploratory pilot study, a sample size calculation was not undertaken.

Two hard-copy surveys were developed, one for dentists and students and another for patients (see appendix). The surveys contained some multiple-choice options but the majority of responses required written answers in the dentist and student surveys. In addition, participant demographic characteristics were collected. Data were collected across four treatment modalities: direct restorative, crowns, dentures, and endodontic treatment. Questions for dentists and students included treatment longevity estimates; free remediation times; reasons for failure covered; and attitudes, length and conditions on warranties. This was a mixed-methods study with questions requiring quantitative and qualitative responses with a mixture of multi-choice and open-ended responses. The patient survey included diagrams and descriptions of each treatment type and questions related to previous treatment experience, and expectations about longevity and free remedial treatment.

All participants were required to read the information sheet and provide written consent prior to taking part in the survey. Ethical approval for the study was obtained from the University of Otago Human Ethics Committee (D21/386). Participant anonymity was maintained as no identifiable data were collected for the purpose of analysis. Data collection occurred in a ten-day working period in July 2021.

Ethnicity group data were cleansed prior to analysis and presented using prioritised ethnicity groups in accordance with Statistics New Zealand prioritisation guidelines [9]. Data were analysed using SPSS v27. No surveys were excluded from the analysis. Missing data were handled using pairwise deletion. Descriptive statistics included means and standard deviations (SD). Statistical evaluation of differences in the mean values according to the participant group (dentist, student, or patient) was performed with Mann-Whitney U tests. Chi-square tests compared dentists’ and students’ willingness to issue a warranty. Pearson correlation tests were used to analyse correlations between patient age and longevity estimates and dentists’ years in practice and longevity estimates, free remediation times, and warranty times. The level of significance was set at *p* < 0.05. Qualitative data were analysed thematically to uncover underlying themes within the responses. This analysis involved a careful, manual coding process, where responses were initially examined to identify key concepts and ideas. These were then systematically organised and iteratively refined into broader themes that accurately captured the primary insights and perspectives expressed by the participants. Our thematic analysis was predominantly inductive, allowing themes to naturally emerge from the data without being constrained by preconceived categories. To illustrate the participants’ viewpoints, representative quotes have been selected.

## Results

### Demographic findings

Overall, 27 students, 28 dentists, and 43 patients responded to the survey. As a result of the convenience sampling method, the response rate was unknown. The mean age of the patients was 47.1 ± 15.4. Table 1 shows participant characteristics. All students were final year dental students.

**Table 1:** Participant characteristics

	Dentist (%)	Student (%)	Patient (%)
**Ethnicity**			
Māori		3 (11.1%)	8 (18.6%)
NZ European	8 (28.6%)	1 (3.7%)	16 (37.2%)
Pacific Islander	1 (3.6%)	6 (22.2%)	11 (25.6%)
Asian	18 (64.3%)	17 (63%)	4 (9.3%)
Other	1 (3.6%)		3 (7%)
Missing			1 (2.3%)
**Gender**			
Male	17 (60.7%)	14 (51.9%)	21 (48.8%)
Female	11 (39.3%)	13 (48.1%)	22 (51.2%)
**Practice**			
Private Practice	24 (85.7%)		
DHB/Community	3 (10.7%)		
University	7 (25%)		
**Years in Practice**			
0–9	12 (42.9%)		
10–19	7 (25%)		
20–29	4 (14.3%)		
30+	5 (17.9%)		
**Total**	28	27	43

### Willingness to issue warranties

**Fig. 1:**
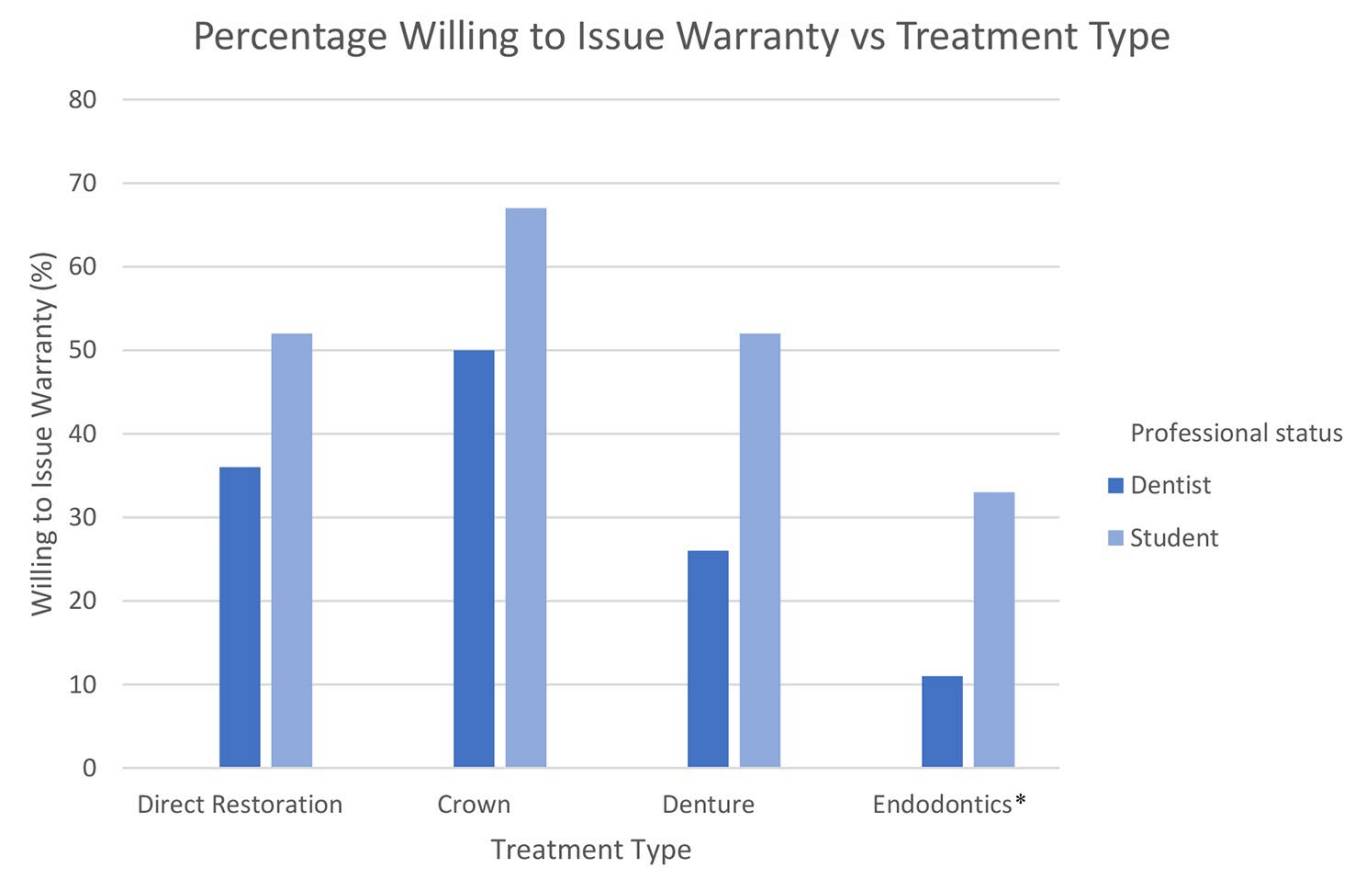
Professional status and willingness to issue warranties for various treatments. *p < 0.05 Chi squared test

The professional status and the willingness to issue warranties for various treatments are shown in Fig. 1. Crowns were perceived to be the most warrantable, followed by direct restorations, dentures and, lastly, endodontics. Significantly, more students were willing to issue a warranty on endodontic treatment than dentists (*p* = 0.04). Binary logistic regression showed age, gender, ethnicity, and years in practice were not significantly associated with willingness to issue a warranty.

### Reasons for free remediation

**Fig. 2:**
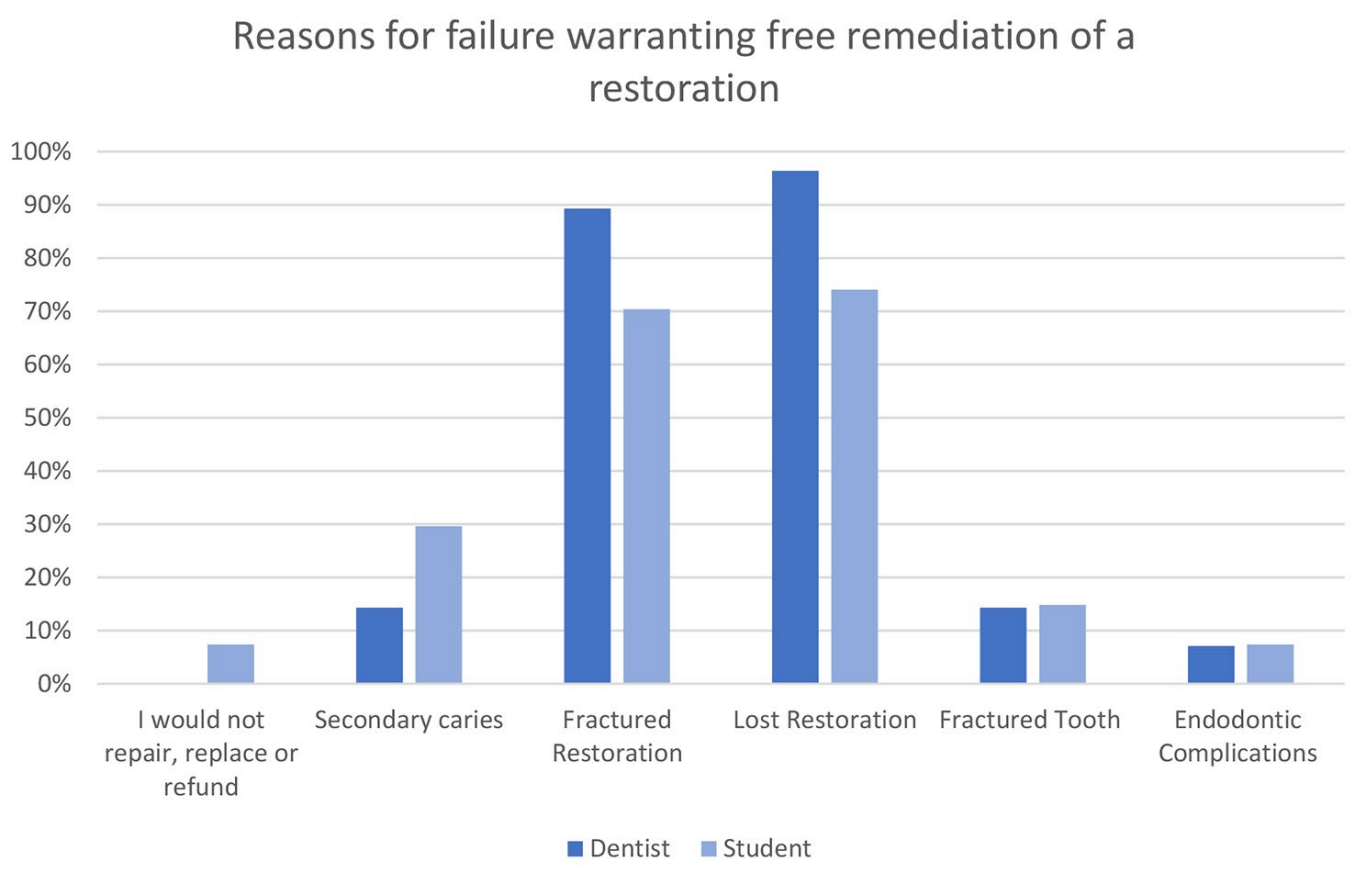
Reasons for failure warranting free repair, replacement or refund of a posterior composite resin restoration by dentists and students

**Fig. 3:**
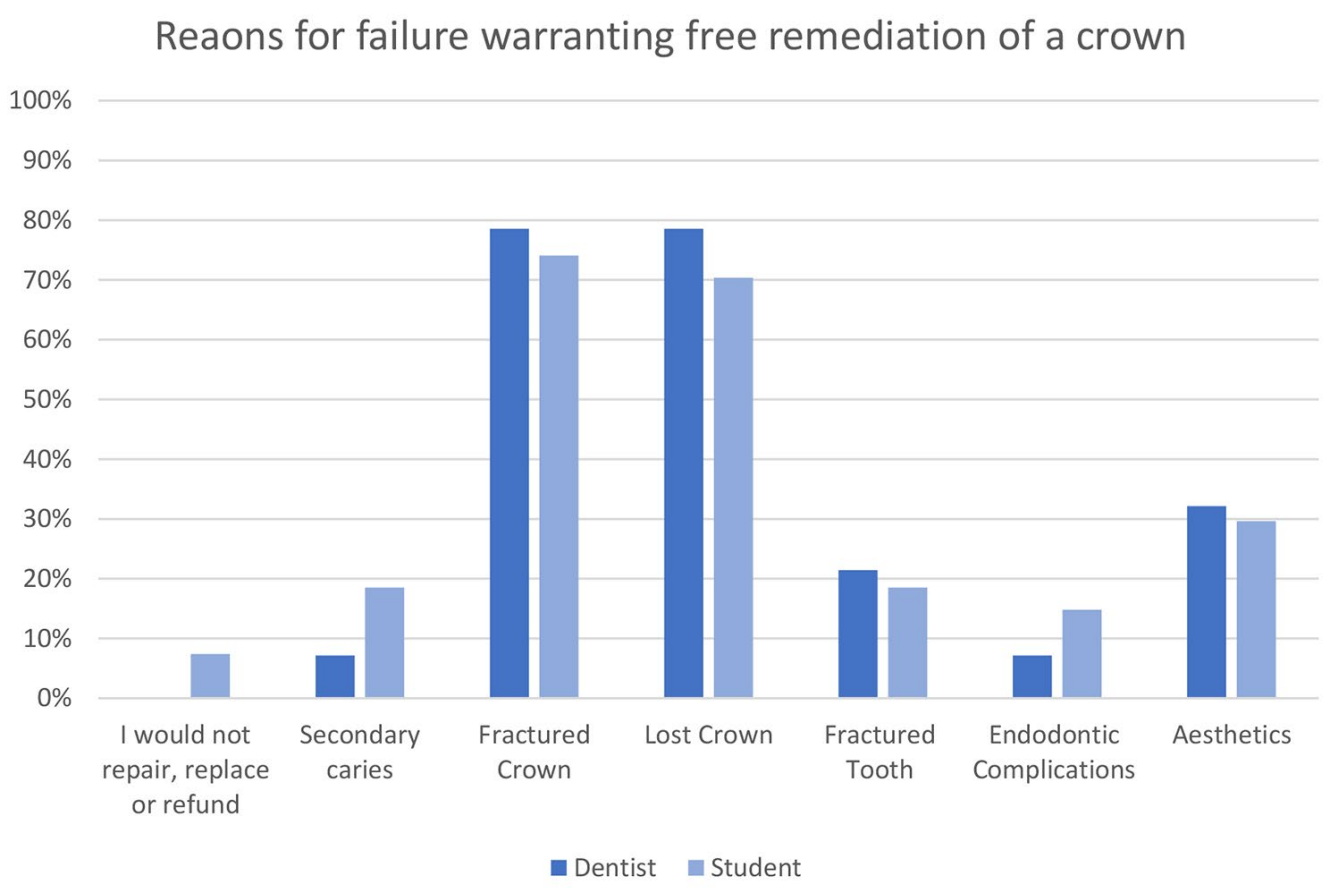
Reasons for failure warranting free repair, replacement or refund of a crown by dentists and students

The reasons for failure that would warrant free repair, replacement or refund of posterior composite resins and crowns are shown in Figs. 2 and 3. Fractured or lost restoration and crowns are the predominant reasons for failure that warrant free remedial treatment or refund.

### Longevity estimates, free remediation and warranty times

The professionals’ views on longevity, free remediation time and warranty period for direct restorations, crowns, dentures and endodontics are shown in Table 2. Only numerical responses were included in this table. Invalid or missing responses were excluded. Although students had lower estimates for posterior composite restoration longevity, there was no significant difference in the mean warranty time. There was no significant difference between dentist and student longevity estimates for crowns, but students’ mean remediation time was significantly lower. The students’ warranty times for crowns were too variable to draw any comparison with the dentists, as they ranged from six months to ten years. Students expected dentures to last nine years, significantly longer than dentists who expected their dentures to last 6.5 years. A Pearson correlation test showed no significant correlation between dentists’ years in practice and longevity estimates, remediation times or warranty times. Most dentists indicated they would remediate failed restorations and crowns, adjust dentures but would not reline dentures or refund for failed endodontics.

**Table 2:** Professional’s views on the longevity, free remediation, and warranty times for various treatment modes

Treatment Mode	Provider	Mean Longevity (months ± SD,n)	Mean Remediation Time^a^ (months ± SD n)	Would Not Remediate^a^ (n)	Mean Warranty Time (months ± SD n)
Posterior Composite	Dentist	80.6 ± 27.8* (28)	16.0 ± 10.7 (25)	(0)	17.0 ± 14.5 (26)
	Student	64.6 ± 24.9* (27)	13.5 ± 12.7 (25)	(2)	19.8 ± 14.3 (27)
Anterior Crown	Dentist	131.5 ± 44.4 (28)	37.2 ± 20.9* (24)	(4)	
	Student	120.8 ± 55.4 (27)	23.9 ± 23.7* (22)	(5)	
Posterior Crown	Dentist	128.5 ± 50.2 (28)	37.5 ± 20.9* (24)	(4)	
	Student	117.7 ± 48.0 (27)	24.4 ± 33.3* (22)	(5)	
Crowns	Dentist				49.1 ± 23.0* (26)
	Student				40.7 ± 61.4* (26)
			Adjustment		
Dentures	Dentist	78.5 ± 36.9* (22)	26.4 ± 76.8 (25)	(1)	20.4 ± 18.1 (21)
	Student	112.4 ± 51.0* (27)	15.4 ± 36.5 (24)	(1)	27.9 ± 22.2 (27)
			Reline		
	Dentist		6.67 ± 4.1 (13)	(14)	
	Student		11.4 ± 9.7 (22)	(9)	
Endodontics	Dentist	102.7 ± 44.9 (25)	13.9 ± 7.2 (15)	(13)	18.3 ± 16.1 (25)
	Student	124.5 ± 73.4 (24)	20.7 ± 19.2 (12)	(15)	19.5 ± 18.8 (25)

*p < 0.05

^a^ defined as free repair, replacement, or refund for restorative and crowns; adjustment or reline for dentures; refund for specialist referral for endodontics

Numerical data for patients’ previous experience, longevity estimates, and free remediation expectations are shown in Table 3. Patients expected posterior fillings to last much longer than dentists and students (*p* < 0.01). There is little variation in longevity estimates across different treatment modalities. Patients expected all dental treatment to last around 11 years. There were a number of missing or invalid responses and non-numerical answers such as ‘don’t know’ or ‘forever’ in the patient questionnaire. The proportion of patients estimating treatment would last forever was 14% for posterior fillings and crowns, 19% for root canal treatment and 21% for dentures. These estimates, as well as missing or invalid responses, were excluded from statistical analysis. A Pearson correlation test was conducted between patient age and the expected longevity of the treatment. There was a moderate positive correlation between patient age and expected longevity of a posterior filling, r(28) = 0.476, *p* = 0.027; and between patient age and expected longevity of a crown r(28) = 0.528, *p* = 0.017. No significant associations were found between patient age and expected longevity of complete dentures or root canal treatment. Binary logistic regression showed age, gender and ethnicity were not significantly associated with an expectation of free remediation.

**Table 3:** Patient’s previous experience, longevity estimates and free remediation expectations

	Had Treatment %	Mean Longevity in months (n)	Expecting Free Remediation^a^ % (n)	Mean Remediation Time in months (n)	
Fillings	100%	131.4 ± 103.9^b^ (31)	74.40% (43)	27.6 ± 41.0 (20)	
Crowns	37.20%	145.7 ± 57.9 (21)	74.40% (41)	34.5 ± 45.0 (20)	
Dentures	18%	133.0 ± 80.5 (23)	30.20% (38)	42.7 ± 58.9 (8)	
Endodontics	60.50%	136.8 ± 64.9 (26)	62.80% (40)	37.7 ± 85.4 (17)	

^a^defined as repair, replacement or refund for fillings, crowns and endodontics, and maintenance for dentures

^b^ Mann Whitney U test of significance compared to professionals *p* < 0.001

### Qualitative findings

#### Direct restorations

There was a general hesitancy about issuing warranties for direct restorative dentistry. Variability in patient factors that influence restoration survival was the main reason cited, but there were a few limited circumstances where it was thought to be more reasonable.

Dentist: “I consider restorative dentistry comparable to a medical device subject to uncontrolled biological complications. But there is always a place for warranty based on material/operator failure”.

The notion of a warranty is better received by students than dentists. Many perceived it to be only fair.

Student: “Warranties should be an integral part of honest and professional dental practice, and patients should be well aware of their right to warrantied goods and services from dentists”.

Although fewer, some dentists agreed.

Dentist: “I think if there was a standard dental warranty, it would increase trust in the profession as a whole and help with the management of patient expectations”.

Several students commented that they felt there needed to be further education in university about the subject of warranties.

When asked what conditions might be placed about a direct restorative warranty, the most common condition listed by dentists related to patient attendance, stating that the patient must adhere to the prescribed examination and hygiene recall period and promptly attend an appointment to report any issues. In contrast, virtually no students felt patient attendance should be a warranty condition. Dentists and students agreed that adequate homecare should be included as a condition and that only certain modes of failure would be included. The consensus was that mechanical failure of the restoration due to operator error would be covered, but failure related to the underlying tooth would not. Dentists and students agreed that if there was a discussion relating to poor prognosis or if an alternative treatment was recommended but declined (i.e. a crown), this treatment would be excluded from warranty. In general, patients with parafunctional habits were excluded from warranty.

#### Crowns

Crowns were perceived to be more warrantable than direct restorative, although similar hesitations apply. Some dentists perceived a warranty on crowns to be more reliable as the material is generally also warranted by the laboratory. A few students commented that a crown warranty would be a good idea as it could be used as a marketing tool, attract more patients, and encourage patients to opt for more expensive treatment. Some students also thought a warranty would be mutually beneficial for the patient and the dentist.

Student: “Good idea as patients will be more willing to spend more money on expensive treatment such as crown & bridge work.”

The same warranty conditions that applied for restorative dentistry were listed for crowns. This included patient attendance, adequate homecare, mechanical failure only, and not for teeth with poor prognoses. A few dentists would only offer a warranty if the providing laboratory also warranted the material.

### Dentures

In general, dentists and students felt that a warranty on dentures was not appropriate. Dentists felt dentures were too difficult to warrant, largely because patients’ abilities to cope with dentures vary.

There are many possible clinical factors which may affect the patient’s satisfaction for dentures.

Students’ viewpoints were more varied, with no consensus. However, a few of the students who left a comment responded positively to the notion.

“More suitable than a crown or restoration because natural tooth structure is not involved.”

When asked to list conditions they would place on a warranty for dentures, dentists, and students felt the mode of failure and attendance at review appointments were most important. However, there was no real consensus about what modes of failure would be covered under warranty. Modes of failure that were discussed included fracture, general wear, and aesthetics. Some participants specifically included these in the warranty, while others explicitly excluded them.

#### Endodontics

Dentists and students were unanimous in their opposition to a warranty for endodontics. They felt that warranting endodontic treatment was risky, commenting on its ‘unpredictability’ as it ‘does not have a 100% success rate.’ The consensus was that the risk of failure should be strongly emphasised during the informed consent process and, therefore, not covered by a warranty. Dentists and students both felt that endodontic treatment is essentially a biological issue and, therefore, it is perceived as the riskiest treatment to warrant. The difficulty of cases and lack of confidence were expressed among students. Dentists commented that they refer difficult cases to specialists.

Dentist: “It is difficult to offer a clear warranty on a biological system.”

Because most dentists and students would never consider a warranty of endodontic treatment, the conditions listed are essentially arbitrary. However, the consensus was that to be eligible for a warranty, a fault must be demonstrated within the initial endodontic treatment, and there would be a requirement for an indirect coronal restoration placed within a prescribed time frame.

## Discussion

### Patient expectation of treatment longevity

This survey found that patients’ longevity estimates were much more optimistic than dentists or students, with a significant proportion ranging from 14 to 21% expecting various dental treatments to last forever. There were significant disparities in expectations of posterior composite resin longevity between patients and practitioners found in the present study. Older patients tended to expect posterior fillings and crowns to last longer. In a study on dental implants, a significant proportion of patients perceived dental implant treatment as life-lasting. A lower education level was associated with some of these misconceptions [10]. Educational level was not investigated in the present study but could be an area of future investigation. In another study on maxillofacial prostheses, optimistic patient expectations were again noted [11]. This highlights some concerning and unrealistic expectations that some patients have around the longevity of dental treatment, which might be sources of dissatisfaction.

### Practitioner expectation of treatment longevity

Dentists and students were asked to estimate how long their treatment would last but were not given a clinical scenario or patient profile, as in a previous study by Maryniuk and Kaplan (1986). In the context of warranties in the present study, this was deemed inappropriate, as a warranty would need to cover a range of scenarios. Patients will inevitably ask their providers how long they should expect treatment to last, but the answer may not be straightforward. Dentist and student longevity estimates are relevant because this should be reflected in their communication with patients. Disparities between practitioner longevity estimates and longevity reported in the literature may result in inaccurate information being relayed to the patient.

The question “how long does treatment last?” is not directly addressed in the literature.

In the search for treatment longevity, meta-analyses often report annual failure rates or success rates for a specified time interval based on Kaplan-Meier survival curves [12–18]. This data is difficult to understand for the patient and does not directly answer the question. Cross-sectional data reports median survival time or ‘clinical longevity time’ (CL_50_), the survival time for 50% of restorations, which may better answer the question, but these study designs have limitations [2, 19–21]. No single parameter directly answers this question. In general, it appears that students and dentists are conservative in their estimates of the longevity of their own treatment. Studies of dentists and dental students show mean and median composite resin survival times of around 7.5-8 years [19, 20, 22]. Dentists and students in the present study expected their posterior composite restorations to last 6.75 and 5.5 years, respectively. Approximately half of all crowns will last 15 years [21] but dentists and students expected the longevity of their crowns to be around 11 years and 10 years, respectively. These estimates are similar to findings in a study of American dentists who estimated their cast restorations to last 12 years [23]. Although 14% of patients expected crowns to last forever, those who accepted the possibility of failure expected crowns to last 12 years, which is comparable to dentists’ expectations.

Students expected dentures to last significantly longer than dentists, 9 years compared to 6.5 years. Compared to a recent meta-analysis, both groups are pessimistic about their denture longevity, as complete dentures had a mean longevity of 10.1 years [3].

Dentists expected their root canal treatments to last 8.5 years, while students expected them to last 10.3 years. Failure was not defined in the present study. There is a distinction between success rates (periapical healing) and survival rates (tooth survival) in the literature. In one study, median survival was 10 years (periapical healing) and 21 years (tooth survival), recognising that a tooth may serve in function for a considerable time despite the presence of a periapical lesion [13]. The hesitancy towards warranting root canal treatment may be partially explained by the inconsistency of the definition of failure in the literature.

It seems, then, that clinicians lack the information to answer the question “how long does it last?” accurately. Perhaps they are better equipped to answer, “how long *should* it last?”. While they might not be able to directly answer how long a treatment will last for a patient, they might have a better idea of the minimum acceptable length of service for their treatment.

Failure for each treatment modality was not defined in the survey and could have been interpreted differently by the participants, resulting in inaccurate longevity estimates. However, in the context of warranties, clinicians would be required to define failure, which was left open for qualitative questioning.

Expected longevity estimates may reflect a clinician’s skills, knowledge, and experience. Patient demographic, socioeconomic and behavioural factors have been demonstrated to influence restoration longevity [24]. Students, compared to dentists, had lower expectations for treatment longevity. This might reflect a lower level of experience and confidence. However, they had higher estimates for the longevity of endodontic treatment and dentures. Dentists’ years in practice were not shown to be associated with longevity estimates. The student perspective may be more influenced by university education. In general, compared to the literature, both dentists and students in our study are pessimistic.

### Expectations for free remediation

Patients expect free remediation for failed fillings, crowns, and endodontic treatment. dentists and students are prepared to meet these expectations within a specified time frame. However, most patients would not expect any free maintenance for dentures. Most dentists and students would adjust dentures for free within a specified time period, but a significant proportion would not reline for free. There is a large discrepancy in expectations for endodontic treatment. If root canal treatment failed and required specialist referral, roughly half of dentists and students would not offer a refund, but almost two-thirds of patients would expect a refund or free remedial treatment. In general, while most clinicians would provide free remediation for treatment that fails within a time frame, the patients appear to disagree about the length of this time frame. However, this difference was not shown to be statistically significant in this study. Remediation types (repair, replace or refund) were pooled into a single category for this study, but it is reasonable to believe that dentists might be more willing to repair or replace failed treatment than they are to refund. Similarly, patient expectations for different remediation types may differ. Further studies could examine patient expectations for differing remediation types.

### Willingness to issue warranties

In general, most dentists are unwilling to issue dental warranties for any treatment. Students are more willing to issue warranties, with half willing to issue warranties for direct restorations and dentures, while two-thirds are willing to issue a warranty for crowns. The difference may be attributed to different practising environments or levels of experience. Students practice in a university environment, where patients pay for low-cost dentistry, and the liability of any free remedial treatment falls on the university. Most dentists in the survey operate in private practice and bear the financial burden of any free remedial treatment covered under warranty.

### Perceptions and conditions on warranty

Crowns, in general, are perceived to be the most warrantable treatment by dentists and students, followed by direct restorations, dentures and, lastly, endodontic treatment. The hesitancy towards offering warranties on dental treatment is due to the variability in factors that influence treatment outcomes, which cannot all be attributed to the dentist. The consensus for direct restorations and crowns is that only mechanical failure of the material would be covered under warranty, and the patient must demonstrate adequate homecare. The most common condition listed by dentists was adherence to examination and hygiene recalls, but students felt this was inconsequential. This may reflect the lack of continuity of care and recall in undergraduate studies. Failures related to the underlying tooth, such as endodontic complications or secondary caries, were typically excluded from warranty. The suggested warranty period by dentists and students is approximately 18 months for restorations. These conditions in an 18-month warranty period would be relatively ‘safe’ for the clinician. The majority of failures that occur within the first year are due to endodontic complications. Recent reviews conclude that caries and fractures are the main reasons for failure, and both of these are typically present in later years [12, 14, 17]. The most common reasons for crown replacement include crown fracture, aesthetics, and secondary caries [25]. Under the most commonly agreed warranty conditions, failure due to crown fracture would be included, although aesthetics and secondary caries are generally excluded.

Dentists felt it was too difficult to warrant dentures because of the variability of a patient’s ability to cope. They may be justified in their hesitance as relatively high failure rates of 5% within the first two years of denture provision may represent lost dentures, manufacturing defects, poor denture design, or immediate dissatisfaction [3]. Ten to fifteen per cent of patients are dissatisfied with new and technically well-made dentures [26].

Only one in ten dentists and a third of students would consider issuing a warranty for endodontic treatment (*p* = 0.04). Therefore, the warranty period and conditions suggested are largely arbitrary. Hypothetically, if dentists and students were to offer a warranty on endodontic treatment, they would only cover failure where fault can be demonstrated in the initial endodontic treatment, and they would have prosthetic requirements. These conditions are reasonable, as intraoperative factors are the primary reasons for the persistence or progression of apical periodontitis [15], and a crown increases the chances of periapical healing and tooth survival [13] while significantly decreasing the chance of tooth fracture [27].

### Limitations

One challenge in writing the questionnaires is posing questions that are simple enough to be understood by the patient but can be compared to the dentist and student survey. Missing data particularly for the patient group, due to misinterpretation, or lack of knowledge around particular dental treatment modalities may introduce bias. A low proportion of patients had previous experience with crowns and dentures, rendering their responses partly invalid. However, these responses might mimic the scenario of a patient receiving that treatment for the first time and provide insight into the expectations of this specific patient group.

Another important limitation was the study’s relatively small sample size, making it difficult to obtain statistically significant data even though a number of trends were detected.

Any generalising from our findings should be done with caution. Dentistry in New Zealand is largely privately funded out-of-pocket, with limited provision for insurance-based payment or publicly funded dentistry. The perspectives on warranties for dentists who provide insurance-based or public-based dentistry require further investigation. Most patients in the study paid out-of-pocket but received heavily subsidised treatments, meaning they may have different expectations from full-fee paying patients in private practice. The opinions of students may also be influenced by this university environment.

Furthermore, the sample was also not representative of dentists and patients in New Zealand. There may be selection bias due to the overrepresentation of female and less experienced dentists. Previous studies have demonstrated that female and less experienced dentists provided shorter longevity estimates [28]. This might explain the conservative longevity estimates from dentists in this study. The ethnic distribution of participants in this study is not representative of the overall New Zealand population, so any generalising about ethnicity should be done cautiously.

Despite these limitations, to the authors’ knowledge, this is the first study investigating perspectives on the notion of a dental warranty. Additionally, few studies have investigated patient expectations around treatment longevity and remediation for failed treatment. Therefore, identifying disparities in the dentist’s and the patient’s expectations, such as restoration longevity and failed root canal remediation, is clinically important as it highlights potential sources of patient dissatisfaction requiring better patient education and communication.

### Future research

One of the strengths of this study is the breadth and large number of variables collected. It highlights several interesting observations that fuel curiosity for further research. Therefore, this study serves as a foundation for future research into dental warranties and patient education. The authors suggest that studies repeat the quantitative element with a larger, more representative sample size, with a revised questionnaire to reduce missing data. Data were not collected separately for remediation types (repair, replace or refund) in this study. It is valid to assume willingness for repair, replacement, and refund would be different. Future research could focus more closely on these types of remediation and analyse them separately with larger sample sizes.

The patient survey should be further simplified to increase valid responses. Patients should be able to answer questions on treatments they have received in the past to increase the relevance of their expectations. Building on the qualitative findings in this study, interviews could be designed to further explore the perspectives on the notion of a dental warranty and understand some of the reasons for disparities. The present study has no qualitative element for patients, and an interview study design is recommended for future studies into patient expectations and perspectives.

## Conclusions

Our study reveals that within New Zealand, dentists generally show reluctance to provide formal warranties on dental treatments, often citing the variability of factors affecting treatment outcomes. Conversely, dental students appear more inclined towards offering warranties. Both groups, however, demonstrate a willingness to offer free remediation or refunds for failed treatments, with the notable exception of root canal procedures. Interestingly, our findings indicate that patients typically hold more optimistic expectations regarding the longevity of dental treatments than both dentists and students, with older patients exhibiting even higher expectations. While these conclusions are primarily applicable to the population receiving dental services at Otago University, they may also provide valuable insights into broader trends in patient expectations and professional attitudes towards dental warranties, which could be relevant in similar healthcare contexts.

### Electronic supplementary material

Below is the link to the electronic supplementary material.

**Supplementary Material 1:** Dentist and student questionnaire

**Supplementary Material 2:** Patient questionnaire

## Data Availability

The datasets used and/or analysed during the current study are available from the corresponding author upon reasonable request.
