# Supplementary material for: Attitudes regarding a warranty and the expected longevity of dental treatment amongst New Zealand dentists, dental students, and patients: a mixed methods survey
Source: BMC Oral Health. 2024 Jan 13;24:74. doi: 10.1186/s12903-024-03860-3 (PMC10787465; doi:10.1186/s12903-024-03860-3)
Supplement: Supplementary file 1 — Supplementary Material 1: Dentist and student questionnaire [file 12903_2024_3860_MOESM1_ESM.pdf]

Perspectives on the notion of dental warranties and expected longevity of dental treatment

1. If you would like to receive a copy of the results, please provide an email address

____

2. What ethnic group do you belong to?

Check all that apply.

- [ ] New Zealand European
- [ ] Māori
- [ ] Pacific Islander
- [ ] Asian
- [ ] European
- [ ] Middle Eastern
- [ ] Latin American
- [ ] African

Other: [ ] ________________________________

3. What year did you graduate or will you graduate as a general dentist?

____

4. What type of practice do you operate from?

Check all that apply.

- [ ] Private Practice
- [ ] DHB/Community
- [ ] University

Other: [ ] ________________________________

5. What is your gender?

Mark only one oval.

- ◯ Male
- ◯ Female
- ◯ Non Binary
- ◯ Prefer not to say
- ◯ Other: ____________________

Restorative Dentistry

6. If you placed a posterior composite resin restoration, how long would you expect it to last?

*Specify if your answer is in months or years - **Do not put a range***

____

7. If a posterior composite restoration failed, within what time frame would you refund, replace or repair it for free?

*Specify if your answer is in months or years - **Do not put a range***

Mark only one oval.

- ◯ ____________________
- ◯ I don't think it should be refunded, replaced or repaired for free

8. For which reasons of failure would you consider repair, replacement or refund of a posterior composite restoration for free?

Check all that apply.

- ☐ I would never consider repair, replacement or refund of a posterior composite restoration for free
- ☐ Secondary Caries
- ☐ Fractured Restoration
- ☐ Lost restoration
- ☐ Fractured tooth
- ☐ Endodontic Complications

Other: ☐ ____________________

9. Would you consider offering a formal dental warranty on restorative dentistry?

Mark only one oval.

- ◯ Yes
- ◯ No

10. Hypothetically, if you had to issue a limited dental warranty on restorative dentistry, how long would the warranty period be? *Specify if your answer is in months or years - **Do not put a range***

____

11. Hypothetically, if you had to issue a limited dental warranty on restorative dentistry, what conditions would you place? (if any)

____
____
____
____
____

12. Do you have any comments on the notion of dental warranties for restorative dentistry?

____
____
____
____
____

13. If you placed a crown how long would you expect it to last?

*Specify if your answer is in months or years - **Do not put a range***

Anterior________

Posterior_______

14. If a crown failed, within what time frame would you replace or repair it for free?

*Specify if your answer is in months or years - **Do not put a range***

- ☐ Anterior_________
- ☐ Posterior_________
- ☐ I don't think it should be replaced or repaired for free

15. For which reasons of failure would you consider repair, replacement or refund of a posterior crown for free?

Check all that apply.

- ☐ I would not repair, replace or refund
- ☐ Secondary Caries
- ☐ Fractured Crown
- ☐ Lost Crown
- ☐ Fractured Tooth
- ☐ Endodontics Complications
- ☐ Aesthetics

Other: ☐ ______________________________

16. Would you consider offering a formal dental warranty on a crown?

Mark only one oval.

- ⬭ Yes
- ⬭ No

17. Hypothetically, if you had to issue a limited dental warranty on a crown, how long would the warranty period be? *Specify if your answer is in months or years – **Do not put a range***

____

18. Hypothetically, if you had to issue a limited dental warranty on a crown, what conditions would you place? (if any)

____

19. Do you have any comments on the notion of dental warranties for crowns?

____

____

____

____

____

Removeable Prosthodontics

20. If you provided upper and lower complete dentures (not immediate), how long would you expect them to last? *Specify if your answer is in months or years - **Do not put a range***

____

21. If you provided upper and lower complete dentures (not immediate) and an adjustment was required (ie for sore spots), within what time frame would you do so for free? *Specify if your answer is in months or years - **Do not put a range***

Mark only one oval.

- ( ) ______________________
- ( ) I don't think it should be adjusted for free

22. If you provided upper and lower complete dentures (not immediate) and a reline was required, within what time frame would you do so for free?

*Specify if your answer is in months or years - **Do not put a range***

Mark only one oval.

- ( ) ____________________
- ( ) I don't think it should be relined for free

23. Would you consider offering a formal dental warranty on complete dentures?

Mark only one oval.

- ( ) Yes
- ( ) No

24. Hypothetically, if you had to issue a limited dental warranty on complete dentures, how long would the warranty period be? *Specify if your answer is in months or years – **Do not put a range***

____

25. Hypothetically, if you had to issue a limited dental warranty on complete dentures, what conditions would you place? (if any)

____

____

____

____

____

26. Do you have any comments on the notion of dental warranties for removable prosthodontics?

____

____

____

27. If you performed root canal therapy, how long would you expect it to last?
*Specify if your answer is in months or years - **Do not put a range***

____

28. If your root canal therapy failed, and you referred to an endodontist, within what time frame would you refund the patient
*Specify if your answer is in months or years - **Do not put a range***

- ( ) ________________________
- ( ) I would not refund the patient

29. Would you consider placing a formal dental warranty on endodontic treatment?

Mark only one oval.

- ( ) Yes
- ( ) No

30. Hypothetically, if you had to issue a limited dental warranty on endodontic treatment, how long would the warranty period be? *Specify if your answer is in months or years - **Do not put a range***

____

31. Hypothetically, if you had to issue a limited dental warranty on endodontic treatment, what conditions would you place? (if any)

____

____

____

____

____

32. Do you have any comments on the notion of dental warranties for endodontic treatment?
