# Supplementary material for: Attitudes regarding a warranty and the expected longevity of dental treatment amongst New Zealand dentists, dental students, and patients: a mixed methods survey
Source: BMC Oral Health. 2024 Jan 13;24:74. doi: 10.1186/s12903-024-03860-3 (PMC10787465; doi:10.1186/s12903-024-03860-3)
Supplement: Supplementary file 2 — Supplementary Material 2: Patient questionnaire [file 12903_2024_3860_MOESM2_ESM.pdf]

How long does dental treatment last?

1. Age

2. Gender

Mark only one oval.

- Male
- Female
- Non Binary
- I'd prefer not to say
- Other:

3. What ethnic group do you belong to? (Mark the space(s) that apply to you)

Check all that apply.

- New Zealand European
- Māori
- Pacific Islander
- Asian
- European
- Middle Eastern
- Latin American
- African

Other:

Fillings

A tooth filling is where material is inserted into a damaged or decayed tooth to restore its original shape and function

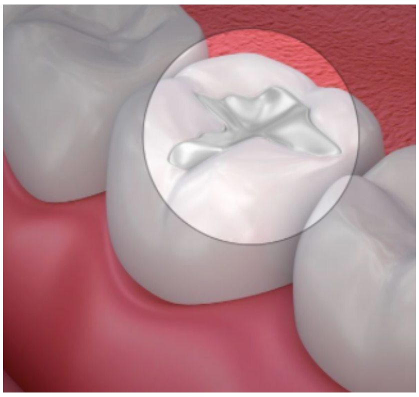

4. Have you ever had a filling?

Mark only one oval.

- Yes
- No
- I don't know

5. If you went to the dentist and got a filling, how long would you expect it to last?

____

6. If your filling failed, would you ever expect a refund, repair or replacement for free?

Mark only one oval.

- Yes
- No

7. If yes, within what time frame would you expect refund, repair or replacement for free?

____

Crown

A dental crown is a tooth-shaped "cap" that is placed over a tooth covering the tooth to restore its shape and size, strength, and/or to improve its appearance.

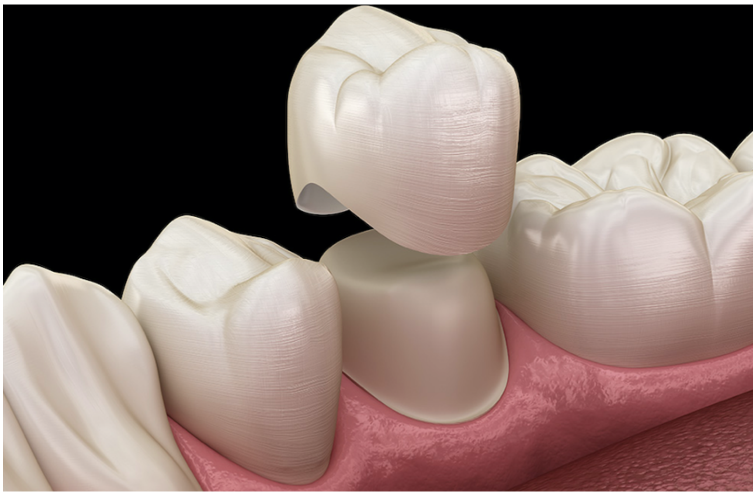

8. Have you ever had a crown?

Mark only one oval.

- Yes
- No
- I don't know

9. If you went to the dentist and got a crown, how long would you expect it to last?

10. If your crown failed, would you ever expect a refund, repair or replacement for free?

Mark only one oval.

- Yes
- No

11. If yes, within what time frame would you expect it to be refunded, repaired or replaced for free?

Dentures

Complete/full dentures are replacements for missing teeth that can be taken out and put back into your mouth.

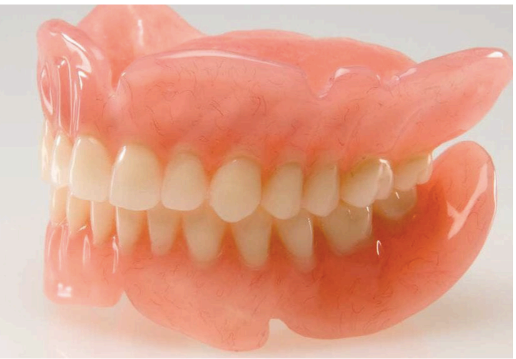

12. Do you wear dentures?

Mark only one oval.

- ( ) Yes- Complete/full dentures
- ( ) Yes- Partial dentures
- ( ) No

13. If you went to the dentist and got complete/full dentures, how long would you expect them to last?

14. If your complete/full dentures required maintenance, would you ever expect it for free?

Mark only one oval.

- ( ) Yes
- ( ) No

15. If yes, within what time frame would you expect maintenance for free?

Root Canal Treatment

Root canal treatment is used to repair and save a tooth where the nerve/pulp is affected by decay or becomes infected.

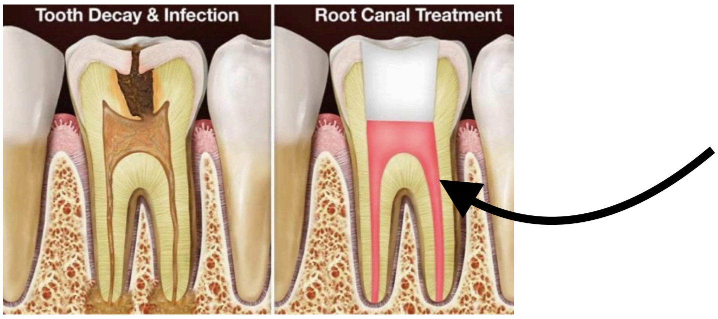

16. Have you ever received root canal treatment?

Mark only one oval.

- ◯ Yes
- ◯ No
- ◯ I don't know

17. If you went to the dentist and got a root canal treatment, how long would you expect it to last?

____

18. If your root canal treatment failed, would you ever expect a refund, repair or replacement for free?

Mark only one oval.

- ◯ Yes
- ◯ No

19. If yes, within what time frame would you expect refund, repair or replacement for free?

____
